# The Role of Non-Selective β-Blockers in Compensated Cirrhotic Patients without Major Complications

**DOI:** 10.3390/medicina56010014

**Published:** 2019-12-30

**Authors:** Wen-Shuo Yeh, Shih-Cheng Yang, Chih-Ming Liang, Yu-Chi Li, Wei-Chen Tai, Chen-Hsiang Lee, Yao-Hsu Yang, Chien-Ning Hsu, Tzu-Hsien Tsai, Seng-Kee Chuah, Cheng-Kun Wu

**Affiliations:** 1 Division of Hepato-Gastroenterology, Department of Internal Medicine, Kaohsiung Chang Gung Memorial Hospital and Chang Gung University College of Medicine, Kaohsiung 83330, Taiwan; email7410@cgmh.org.tw (W.-S.Y.); d5637700@cgmh.org.tw (S.-C.Y.); gimy54861439@gmail.com (C.-M.L.); e3851171@cgmh.org.tw (Y.-C.L.); luketai1019@gmail.com (W.-C.T.); chuahsk@seed.net.tw (S.-K.C.); 2 Division of Infectious Diseases, Department of Internal Medicine, Kaohsiung Chang Gung Memorial Hospital and Chang Gung University College of Medicine, Kaohsiung 83330, Taiwan; lee900@cgmh.org.tw; 3 Department of Traditional Chinese Medicine, Chiayi Chang Gung Memorial Hospital, Chiayi 61363, Taiwan; r95841012@ntu.edu.tw; 4 Health Information and Epidemiology Laboratory of Chang Gung Memorial Hospital, Chiayi 61363, Taiwan; 5 School of Traditional Chinese Medicine, College of Medicine, Chang Gung University, Taoyuan 33302, Taiwan; 6 Department of Pharmacy, Kaohsiung Chang Gung Memorial Hospital, Kaohsiung 83330, Taiwan; cnhsu@cgmh.org.tw; 7 School of Pharmacy, Kaohsiung Medical University, Kaohsiung 80700, Taiwan; 8 Division of Cardiology, Department of Internal Medicine, Kaohsiung Chang Gung Memorial Hospital and Chang Gung University College of Medicine, Kaohsiung 83330, Taiwan; garytsai@adm.cgmh.org.tw

**Keywords:** cirrhotic patients without major complications, clinically significant portal hypertension, propranolol

## Abstract

*Background and Objectives*: Non-selective β-blockers (NSBB) could prevent decompensation and hepatocellular carcinoma (HCC) in cirrhotic patients with clinically significant portal hypertension (CSPH), but remained uncertain for compensated cirrhotic patients without major complications. We aimed to compare the clinical outcomes between propranolol users and non-users of a CC group without major complications. *Material and Methods*: We conducted this population-based cohort study by using the Taiwanese Longitudinal Health Insurance Database 2000. Propranolol users (classified as cumulative defined daily dose (cDDD)) and non-PPL users were matched with a 1:1 propensity score in both cohorts. *Results*: This study comprised 6896 propranolol users and 6896 non-propranolol users. There was no significant impact on the development of spontaneous bacterial peritonitis between the two groups (aHR: 1.24, 95% confidence interval (CI): 0.88~1.75; *p* = 0.2111). Male gender, aged condition, and non-liver related diseases (peripheral vascular disease, cerebrovascular disease, dementia, pulmonary disease, and renal disease) were the independent risk factors of mortality. PPL users had significantly lower incidence of HCC development than non-users (aHR: 0.81, *p* = 0.0580; aHR: 0.80, *p* = 0.1588; and aHR: 0.49, *p* < 0.0001 in the groups of 1–28, 29–90, and >90 cDDD, respectively). *Conclusion*: The current study suggested that high cumulative doses of propranolol could decrease the risk of hepatocellular carcinoma among compensated cirrhotic patients without major complications. Further large-scale prospective studies are still required to confirm the findings in this study. *Results*: It remained uncertain whether non-selective β-blockers (NSBB) could prevent decompensation and hepatocellular carcinoma (HCC) in compensatory cirrhotic patients without major complications. This study aimed to compare the clinical outcomes between propranolol users and non-users of the CC group without major complications.

## 1. Introduction

Portal hypertension (PHT) is the driving force of clinical progression in patients with liver cirrhosis. Non-selective β-blockers (NSBB), available as propranolol in Taiwan, can effectively reduce PHT by the mechanism of reducing the splanchnic blood flow and lowering the cardiac output [1]. Current practice guidelines recommend the use of NSBB as a primary and secondary prophylaxis strategy for cirrhotic patients with presence of esophageal varices (EV) [2,3]. Villanueva C, et al. [4,5] reported that cirrhotic patients with the development of clinically significant portal hypertension (CSPH) had a greater hepatic vein pressure gradient (HVPG) reduction after NSBB treatment than those without CSPH. NSBB could prevent decompensation in cirrhotic patients with CSPH. Apart from this, many studies focused on the use of NSBB in a decompensated group. Some studies reported that NSBB was not associated with increased mortality among decompensated cirrhotic patients with ascites [6,7,8,9], whereas Kalambokis GN, et al. [10] found that an increased mortality was observed in Child-Pugh C cirrhotic patients with ascites if using NSBB for more than six months. Moreover, NSBB could reduce cancer risk [11,12], including hepatocellular carcinoma (HCC) [13,14]. The issue about the use of NSBB on the prognosis in compensated cirrhotic patients without major complications has seldom been reported.

Therefore, we conducted a large population-based cohort study in a national health care setting in an attempt to clarify the clinical impacts of NSBB on cirrhotic patients without major complications.

## 2. Methods

### 2.1. Compliance with Ethical Requirements

The study protocol was approved by the Institutional Review Board and the Ethics Committee of Chang Gung Memorial Hospital at Taoyan in Taiwan (permitted number 201800318B0C503 on 1st April 2019). The Ethics Committee waived the requirement for informed consent for this study, and the data were analyzed anonymously.

### 2.2. Data Sources

The present study analyzed data extracted from the Longitudinal Health Insurance Database 2000 (LHID 2000) of one million individuals (approximately 5% of the entire Taiwan population) who were randomly sampled from the year 2000 Registry for Beneficences of 23.75 million individuals involved in Taiwan’s National Health Insurance (NHI) program [15]. Taiwan’s National Health Insurance program was initiated in 1995, and covers over 99% of Taiwan’s 23 million individuals. LHID 2000 contains the demographic information, diagnostics, medical treatments, prescriptions, and total costs from 1 January 1997 to 31 December 2013.

### 2.3. Study Cohort, and Inclusion and Exclusion Criteria

**Figure 1:**
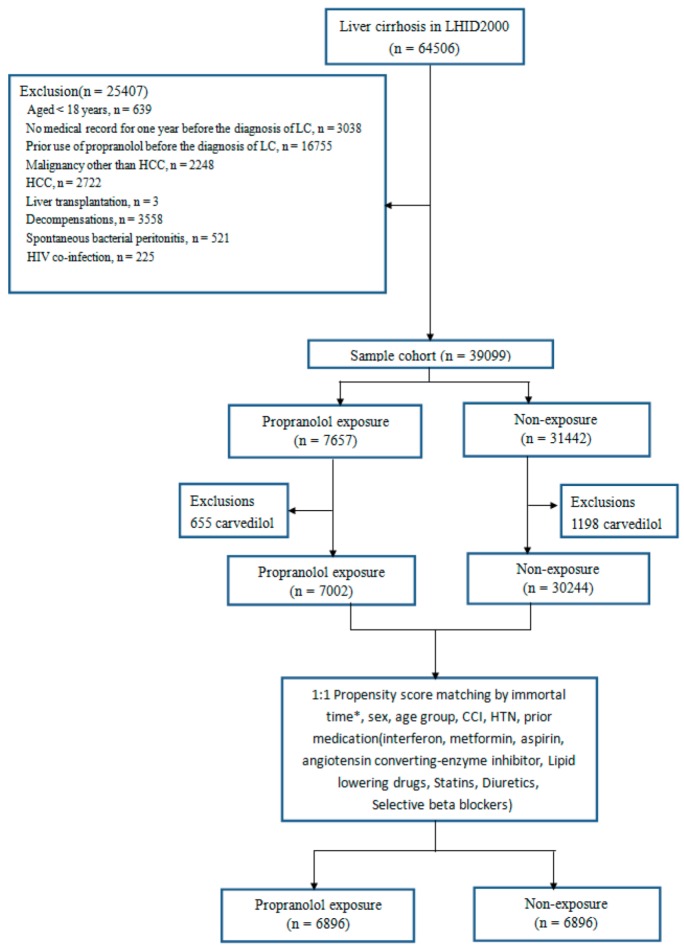
Schematic flowchart of the study design.

Figure 1 shows a schematic flowchart of the study design. The cohort of patients with liver cirrhosis was identified using ICD-9 CM (International Classification of Diseases, Ninth Revision, Clinical Modification, codes: 571.2, 571.5, or 571.6, based on ≥1 claim of inpatients or ≥2 claims of outpatients in one year and apart ≥28 days) between 1997 and 2013. Patients with ≥18 years old were enrolled in the study. The etiology of cirrhosis was collected, namely: chronic hepatitis B virus (ICD-9 CM codes: 070.2, 070.22, 070.23, 070.3, 070.32, 070.33, and V02.61), chronic hepatitis C virus (ICD-9 CM codes: 070.51, 070.54, and V02.62), and alcohol-related disease (ICD-9 CM codes: 291, 303.0, 303.9, 305.0, 571.0, 571.2, and 571.3). In the present study, we focused on only cirrhotic patients without major complications, and with a later stage of chronic liver disease with the development of portal hypertension. Those who had a diagnosis of spontaneous bacterial peritonitis (SBP; ICD-9-CM code: 567), variceal bleeding (ICD-9-CM codes: 456.8, 456.0, and 456.20), ascites (ICD-9-CM code: 7895, or with frequent abdominal tapping, 54.91, based on ≥3 claims of inpatients in one year or ≥1 claims of admission), jaundice (ICD-9-CM code: 7824), hepato-renal syndrome (ICD-9-CM code: 5724), hepatic coma (ICD-9-CM code: 5722), and other sequelae of chronic liver disease (ICD-9-CM code: 572.8) were defined as having a decompensated status and were excluded from the analyses. Accordingly, patients with cirrhosis without any of the above conditions were considered to have liver cirrhosis without major complications. Those who were <18 years old, had a history of Human Immunodeficiency Virus (HIV) co-infection (ICD-9-CM codes: 042, 044, and V08), hepatocellular carcinoma (ICD-9-CM code: 155), malignancy other than HCC (ICD-9-CM codes: 140–208, with major illness certificate), loss of medical record for one year, or prior use of PPL before enrollment were also excluded. After propensity score matching, 6896 patients with propranolol exposure and 6896 patients without exposure were analyzed.

### 2.4. Definition of PPL Exposure

We defined the exposure of propranolol (ATC code: C07AA05) from the diagnosis of liver cirrhosis to the occurrence of outcomes or to the end of follow-up. To further recognize the possible influence of the dose effect, we adopted the concept of quantifying a prescribed dose of medication, anticipating the average prescription dose per day in adult population, which was known as a cumulative defined daily dose (cDDD) [16]. We classified the propranolol dose into four sets in each group (0, 28, 29–89, and >90 cDDDs). Patients were considered as not taking any propranolol if the cDDD was zero. Moreover, some concomitant drugs with potential confounding effects, including selective β-blockers (ATC code: C07AB), diuretics (ATC code: C03), thiazides (ATC code: C03A-C) potassium-sparing drugs (ATC code: C03D), and diuretics and potassium-sparing agents in combination (ATC code: C03E) were identified from the index date to the event of interest or to the end of follow-up.

### 2.5. Study Outcomes

The primary outcome was the development of hepatocellular carcinoma. All of the patients were followed from the index date to the event of interest, or to the end of the three-year follow-up. The secondary outcome was spontaneous bacterial peritonitis, all-cause mortality, liver transplantation (ICD9 code: V42.7), or any diagnosis indicative of decompensation, which is defined as above.

### 2.6. Confounder Assessment

The patients’ underlying comorbid conditions were identified within one year prior to the index date. The burden of comorbid illness was assessed based on the Deyo modification of the Charlson comorbidity index (CCI), which has been shown to be a well-validated measure of comorbidity, adjusting for disease burden in the administrative data [17]. To better understand the comorbid illness on the confounding impact on the development of HCC, the burden of comorbid illness was further separately discussed.

Other potential risk factors, including the liver disease treatment (statins (atorvastatin ATC codes: C10AA05, C10BX08, C10BX03, C10BA05, C10BX12, C10BX06, and C10BX11; fluvastatin ATC code: C10AA04; pitavastatin ATC code: C10AA08; rosuvastatin ATC codes: C10AA07, C10BX05, 10BX09, C10BA06, C10BX10, and C10BX07; simvastatin ATC codes: C10AA01, C10BX01, C10BA02, C10BA04, C10BX04, and A10BH51) [18,19], lipid-lowering agents (clofibrate ATC code: C10AB01; bezafibrate ATC code: C10AB02; gemfibrozil ATC code: C10AB04; fenofibrate ATC code: C10AB05; nicotinic acid ATC code: C04AC01; and acipimox ATC code: C10AD06), angiotensin converting-enzyme inhibitor (captopril ATC code: C09AA01; lisinopril ATC code: C09AA03; perindopril ATC code: C09AA04; ramipril ATC code: C09AA05; quinapril ATC code: C09AA06; benazepril ATC code: C09AA07; cilazapril ATC code: C09AA08; and fosinopril ATC code: C09AA09), aspirin (ATC code: B01AC06), metformin (ATC code: A10BA02), diuretics (furosemide ATC code: C03CA01, C02LA01; spironolactone ATC code: C03DA01), and selective β-blockers (ATC code: C07AB)), were assessed in the study. Only patients with drug use for at least 28 days within one year prior to the index hospitalization, or use for at least 28 days during the study follow-up were analyzed.

### 2.7. Statistical Analysis

We performed a matched case control study by using propensity score adjustment with immortal time, sex, age group, CCI, HTN, and prior medication (interferon, metformin, aspirin, angiotensin converting-enzyme inhibitor, lipid lowering drugs, statins, diuretics, and selective beta blockers). Continuous data were presented as means ± standard deviation (SD), and categorical data were presented as frequencies and percentages. Pearson’s chi-square or Fisher’s exact two-tailed tests were used for the analysis of categorical data, while continuous variables were analyzed using the t-test, where appropriate.

To assess the impact of the competing risk of death during the index hospitalization on the outcome prediction, the standard and cause-specific approach of the Cox proportional hazard model was employed to estimate the relative hazard ratio of an outcome event. Adjustments of patient demographics, clinical conditions, and medication usage were made in the regression model. The Kaplan–Meier method with the log-rank test was employed to compare the cumulative incidence between comparison groups. Two-tailed *p*-values of <0.05 were considered statistically significant. All of the statistical analyses were conducted using SAS version 9.4 (SAS Institute’s Inc., Cary, NC, USA, 2013).

## 3. Results

### 3.1. Patient Characteristics

The baseline characteristics of the parameters before matching are presented in Supplementary Table S1. Table 1 shows the demographic data for the two groups after propensity score adjustment, which included the etiologies of liver cirrhosis, such as hepatitis B virus infection, hepatitis C virus infection, and alcoholism.

### 3.2. Outcomes of Spontaneous Bacterial Peritonitis and Assessments of Risk Factors

**Figure 2:**
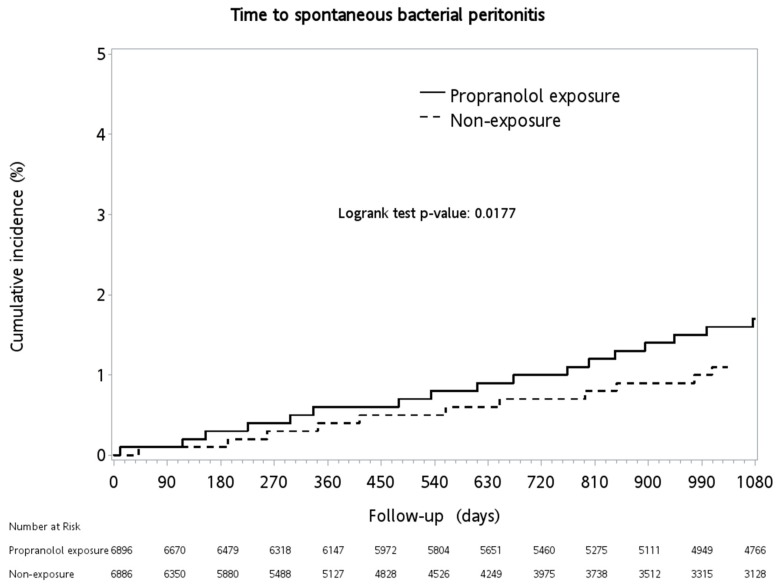
Cumulative incidence of spontaneous bacterial peritonitis between the groups.

As shown in Table 2, patients with propranolol exposure had significantly higher rates of spontaneous bacterial peritonitis (1.42% vs. 0.75%, *p* = 0.0002) during the three-year follow-up period (log-rank *p* = 0.0177, shown in Figure 2) and for ascites (4.32% vs. 0.64%, *p* < 0.0001). No significant difference was observed with respect to hepatorenal syndrome (0.26% vs. 0.15%, *p* = 0.1302).

As shown in Table 3, male gender (aHR: 1.90, 95% CI: 1.30~5.76, *p* = 0.0008), aged ≥65 (aHR: 2.02, 95% CI: 1.08~3.75, *p* = 0.0270), and concomitant diuretic usage (aHR: 8.56, 95% CI: 5.93~12.37, *p* < 0.0001) were independent risk factors for the development of SBP on the multivariate analysis. On the contrary, the concomitant use of selective β-blockers was a protective factor from SBP (aHR: 0.35, 95% CI: 0.19~0.67, *p* = 0.0014). Notably, there was no significant impact on the development of SBP about the exposure of propranolol or not (aHR: 1.24, 95% CI: 0.88~1.75; *p* = 0.2111).

### 3.3. All-Cause Mortality

**Figure 3:**
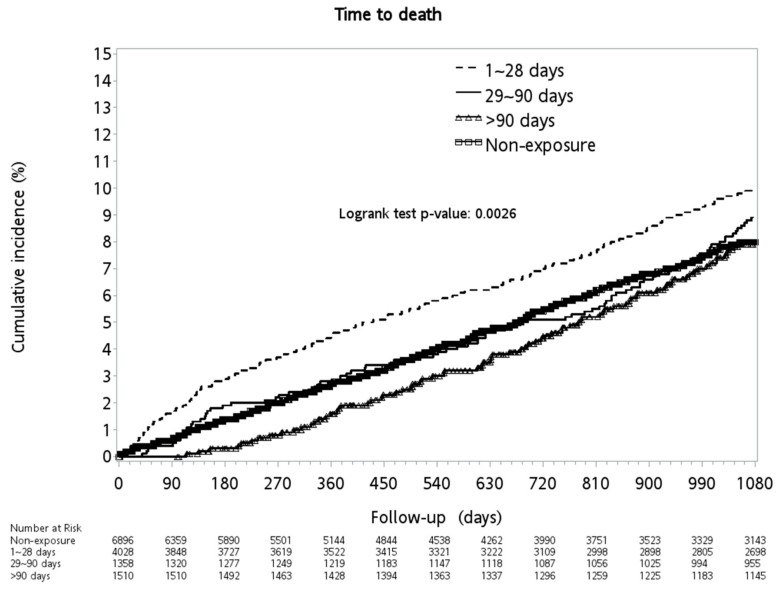
Cumulative incidence of all-cause mortality between the groups.

During the three-year follow-up period, cirrhotic patients without major complications, who had PPL exposure for more than 90 days, had a significantly lower rate of all-cause mortality than those without exposure. Notably, those who had exposure between 1~28 days and 29~90 days had a higher all-cause mortality rate (Figure 3).

As shown in Table 4, on the multivariate analysis, male gender (aHR: 1.59, 95% CI: 1.38~1.83, *p* < 0.0001), an aged condition (35–49 aHR: 1.73, 95% CI: 1.34~2.21; 50–64 aHR: 2.63, 95% CI: 2.04~3.40; ≥65 aHR: 5.07, 95% CI: 3.89~6.61, *p* < 0.0001), peripheral vascular disease (aHR: 3.15, 95% CI: 1.67~5.93, *p* = 0.0004), cerebrovascular disease (aHR: 1.56, 95% CI: 1.20~2.01, *p* = 0.0008), dementia (aHR: 1.86, 95% CI: 1.04~3.32, *p* = 0.0350), pulmonary disease (aHR: 1.25, 95% CI: 1.03~1.52, *p* = 0.0218), peptic ulcer (aHR: 1.29, 95% CI: 1.11~1.50, *p* = 0.0009), renal disease (aHR: 1.87, 95% CI: 1.42~2.48, *p* < 0.0001), baseline (aHR: 1.88, 95% CI: 1.45~2.44, *p* < 0.0001), and concomitant diuretics (aHR: 2.69, 95% CI: 2.33~3.11, *p* < 0.0001) were independent risk factors of all-cause mortality. Baseline Angiotensin-converting enzyme inhibitor ACEI (aHR: 0.71, 95% CI: 0.52~0.97, *p* = 0.0307) and concomitant selective beta blockers (aHR: 0.44, 95% CI: 0.35~0.56, *p* < 0.0001) were protective factors. As for propranolol, a trend from a harmful effect if cDDD was <90 days (1~28 cDDD, aHR: 1.33, 95% CI: 1.15~1.53, *p* = 0.0001; 29~90 cDDD, aHR: 1.12, 95% CI: 0.91~1.40, *p* = 0.2888), to a protective effect if cDDD was more than 90 days (aHR: 0.79, 95% CI: 0.64~0.98, *p* = 0.0340) was observed.

### 3.4. Outcomes of Hepatocellular Carcinoma and Assessments of Risk Factors

In the multivariate analysis, the independent risk factors of HCC development were gender (aHR: 1.47, 95% CI: 1.20~1.79, *p* = 0.0002), aging (35–49 aHR: 3.33, 95% CI: 2.02~5.49; 50–64 aHR: 7.67, 95% CI: 4.68~12.55; ≥65 aHR: 10.72, 95% CI: 6.43~17.88, *p* < 0.0001), and concomitant use of diuretics (aHR: 5.65, 95% CI: 4.61~6.92, *p* < 0.0001). The concomitant use of selective beta blockers was a protective factor (aHR: 0.54, 95% CI: 0.39~0.74, *p* = 0.0001).

**Figure 4:**
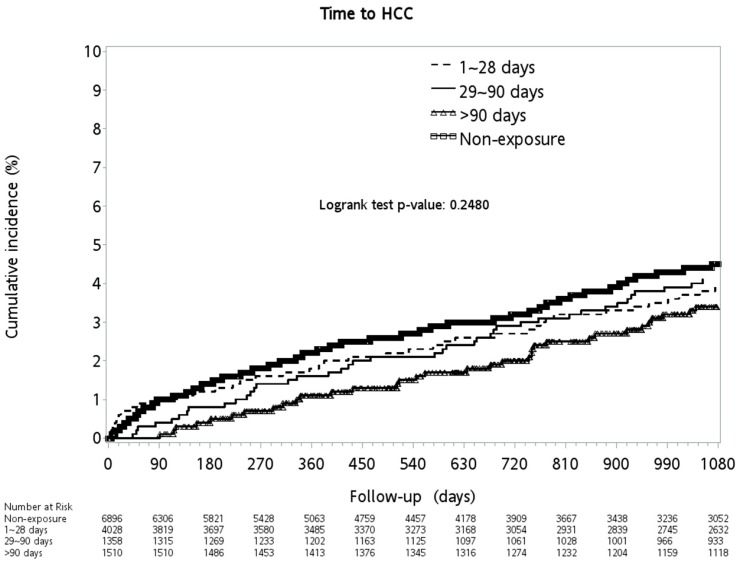
Cumulative incidence of hepatocellular carcinoma between the groups.

As for propranolol, the cirrhotic patients without major complications had an increased protective effect free from the development of HCC when they had an increased cDDD of propranolol exposure (Figure 4 and Table 5). The protective effect was even more significant among those with propranolol exposure for more than 90 days when compared with those without propranolol exposure (aHR: 0.49, 95% CI: 0.36~0.67, *p* < 0.0001).

## 4. Discussions

Clinically significant portal hypertension was defined as a hepatic venous pressure gradient (HVPG) of ≥10 mmHg. Compensated cirrhotic patients with CSPH may present with the appearance of varices and a risk of decompensation [20,21]. Owning to the development of hyperdynamic circulation in cirrhotic patients with CSPH, NSBB could effectively reduce HVPG, and hence improve the clinical outcomes, including bleeding and mortality [22]. On the contrary, compensated cirrhotic patients without CSPH were not suggested the use of NSBB because of the lack of development of hyperdynamic circulation. This current study focused on patients with a diagnosis of cirrhosis, and excluded any diagnosis of decompensation, which meant that we enrolled cirrhotic patients without CSPH clinically. We found that patients with PPL exposure had significantly higher rates of decompensation events than those without PPL (Table 2). It did not mean that PPL caused worse outcomes among cirrhotic patients without CSPH. As shown in Table 1, patients with propranolol exposure still had more comorbidity than those without exposure, even after propensity score matching adjustment. The bottom line was that, in real world practice, the clinical physicians prescribed propranolol to patients with a more serious clinical condition, including a higher trend toward CSPH and decompensations events. The use of NSBB was not helpful in preventing further progress of decompensation among cirrhotic patients without CSPH (Supplementary Figure S1 and Supplementary Table S2).

Most cirrhotic patients suffered from death after the development of decompensation. Many studies focused on the discussion of the safety concerns of NSBB use among decompensated cirrhotic patients, especially with presence of ascites, but they have not been conclusive to date [6,7,8,9,10]. The NSBB might not affect the mortality, but should be used cautiously, especially among enrolled patients with a history of spontaneous bacterial peritonitis [23]. On the contrary, non-liver-related diseases were predominant causes of mortality among compensated cirrhotic patients [24,25]. Similarly, the current study showed that host factors (male gender and aged condition) and comorbidity (peripheral vascular disease, cerebrovascular disease, dementia, pulmonary disease, and renal disease) were the independent risk factors of mortality. Moreover, a protective effect from mortality was observed among patients with NSBB exposure for more than 90 days cDDD (aHR: 0.79, 95% CI: 0.64~0.98, *p* = 0.0340). Brito-Azevedo A, et al. reported that improving endothelial function was detected among compensated cirrhotic patients receiving PPL compared with those without propranolol use (propranolol users, n = 6, 567 ± 377% vs. non-propranolol users, n = 14, 490 ± 188%; *p* = 0.01) [26]. Moreover, propranolol could reduce inflammation by decreasing intestinal permeability, bacterial translocation, and serum levels of IL-6 [27,28], and therefore might explain the dose-dependent effect of PPL on the impact of mortality.

Ripoll C, et al. reported that cirrhotic patients with CSPH had a higher annual incidence of HCC than those without CSPH (2.1% vs. 0.35%) [29]. Furthermore, propranolol had anti-cancer effects and could block the β-2 adrenergic receptor (ADRB2), whose expression was upregulated in HCC [30]. A meta-analysis by Thiele M, et al. reported that NSBB may prevent HCC in patients with cirrhosis [14]. However, those trials enrolled in this meta-analysis were not targeted for the survey of NSBB and HCC. Herrera l, et al. performed a retrospective cohort study including 73 patients treated with NSBB and 100 patients without NSBB use, and found that a lower cumulative incidence of HCC during five or ten years of follow-up was observed among propranolol users. It was reported that increasing the protective effect from the development of HCC was observed among patients with propranolol users (1~28 cDDD aHR: 0.81→29~90 cDDD aHR: 0.80→>90 cDDD aHR: 0.49, as shown in Table 5) compared with those without exposure. The strength of this study was that it was a large sample size population-based study. To our knowledge, it is the first study to demonstrate the quantification of PPL exposure to the association of HCC among cirrhotic patients without CSPH.

Increased vascular resistance was the predominant mechanism of PHT in the early phase of cirrhosis. Carvedilol could decrease the vascular resistance by its intrinsic vasodilator activity, and was reported to be more effective in reducing PHT than propranolol [31,32]. Current practice guidelines recommend carvedilol as the choice of primary prevention for cirrhotic patients with esophageal varices [2]. In this study, we found that patients with a concomitant use of selective BB had beneficial effects for the prevention of SBP, better survival rates, and lower rates of HCC development. For patients with intolerances or non-responders to NSBB, carvedilol might be a promising therapy for compensated cirrhotic patients without CSPH.

This study has several limitations. First, to select compensated cirrhotic patients without CSPH, we performed rigorous exclusions of the diagnosis supportive of decompensation clinically. We could only define the study cohort as a majority of patients with compensated cirrhosis without major complications, but not as compensated patients without CSPH. Non-invasive exams highly suggestive of CSPH, including liver stiffness (≥20–25 kPa by transient elastography) [33] or the detection of porto-systemic shunting or varices [3,33] should be collected in future studies. Second, we could not accurately classify the patients clinically because of the lack of hemodynamics and laboratory data. Propensity score matching adjustment was performed for the correction of potential confounding factors. Third, the actual dose of PPL and the duration were not available from the NHIRD. In this current study, a positive dose-response effect of PPL on the prevention of HCC by using the concept of cDDD was observed. Based on this important finding, a prospective study focused on the dose and the duration of PPL use among compensated cirrhotic patients without CSPH should be further clarified.

In conclusion, the current study suggested that high cumulative doses of propranolol could decreased the risk of hepatocellular carcinoma among compensated cirrhotic patients without major complications. Further large-scale prospective studies are still required to confirm the finding in this study.

## Figures and Tables

**Table 1:** Patient’s characteristics after propensity score matching adjustment.

Variable			Exposure		Non-Exposure			
		N	n	(%)	n	(%)	SMD	*p*-Value
**Total**		13,792	6896	(50.00)	6896	(50.00)		
**Sex**								0.8196
	Female	5237	2625	(38.07)	2612	(37.88)	0.00	
	Male	8555	4271	(61.93)	4284	(62.12)	0.00	
**Age group**							0.01	0.7577
	18–34	3254	1609	(23.33)	1645	(23.85)		
	35–49	5463	2722	(39.47)	2741	(39.75)		
	50–64	3461	1755	(25.45)	1706	(24.74)		
	65+	1614	810	(11.75)	804	(11.66)		
**Covariate**								
	Acute myocardial infarction	12	9	(0.13)	3	(0.04)	0.03	0.0831
	Congestive heart failure	111	67	(0.97)	44	(0.64)	0.04	0.0284
	Peripheral vascular disease	32	18	(0.26)	14	(0.20)	0.01	0.4790
	Cerebral vascular accident	369	184	(2.67)	185	(2.68)	0.00	0.9579
	Dementia	37	20	(0.29)	17	(0.25)	0.01	0.6214
	Pulmonary disease	876	449	(6.51)	427	(6.19)	0.01	0.4424
	Connective tissue disorder	99	52	(0.75)	47	(0.68)	0.01	0.6140
	Peptic ulcer	2194	1113	(16.14)	1081	(15.68)	0.01	0.4563
	Liver cirrhosis	3447	1964	(28.48)	1483	(21.51)		<0.0001
	Hepatitis B Virus	7885	3605	(52.28)	4280	(62.06)		<0.0001
	Hepatitis C Virus	3079	1653	(23.97)	1426	(20.68)		<0.0001
	Alcohol	3544	2159	(31.31)	1385	(20.08)		<0.0001
	Diabetes	920	485	(7.03)	435	(6.31)	0.03	0.0879
	Diabetes complications	189	115	(1.67)	74	(1.07)	0.05	0.0027
	Paraplegia	36	18	(0.26)	18	(0.26)	0.00	1.0000
	Renal disease	250	139	(2.02)	111	(1.61)	0.03	0.0739
	Severe liver disease	3	2	(0.03)	1	(0.01)	0.01	0.5637
	Hypertension	1969	1032	(14.97)	937	(13.59)	0.04	0.0208
**Prior medications**								
**Interferon-based therapy**								
	interferon	43	23	(0.33)	20	(0.29)	0.01	0.6468
**Metformin**		605	328	(4.76)	277	(4.02)	0.04	0.0340
**Aspirin**		520	286	(4.15)	234	(3.39)	0.04	0.0201
**Angiotensin converting-enzyme inhibitor**		376	200	(2.90)	176	(2.55)	0.02	0.2095
	Captopril	136	70	(1.02)	66	(0.96)		0.7303
	Lisinopril	63	35	(0.51)	28	(0.41)		0.3767
	Perindopril	51	25	(0.36)	26	(0.38)		0.8884
	Ramipril	42	23	(0.33)	19	(0.28)		0.5365
	Quinapril	26	13	(0.19)	13	(0.19)		1.0000
	Benazepril	3	2	(0.03)	1	(0.01)		0.5637
	Cilazapril	17	8	(0.12)	9	(0.13)		0.8082
	Fosinopril	62	36	(0.52)	26	(0.38)		0.2031
**Lipid lowering drugs**		222	129	(1.87)	93	(1.35)	0.04	0.0149
	Clofibrate							0.0149
	Bezafibrate	30	13	(0.19)	17	(0.25)		0.4647
	Gemfibrozil	129	78	(1.13)	51	(0.74)		0.0169
	Fenofibrate	74	44	(0.64)	30	(0.44)		0.1027
	Nicotinic acid	4	2	(0.03)	2	(0.03)		1.0000
	Acipimox	7	6	(0.09)	1	(0.01)		0.0587
**Statins**		81	48	(0.70)	33	(0.48)	0.03	0.0946
	Atorvastatin							0.0946
	Fluvastatin	47	26	(0.38)	21	(0.30)		0.4650
	Pitavastatin							0.4650
	Rosuvastatin	30	19	(0.28)	11	(0.16)		0.1437
	Simvastatin	7	5	(0.07)	2	(0.03)		0.2567
**Diuretics**		272	145	(2.10)	127	(1.84)	0.02	0.2703
	Furosemide	233	125	(1.81)	108	(1.57)		0.2613
	Spironolactone	75	36	(0.52)	39	(0.57)		0.7283
**Selective beta blockers**		577	293	(4.25)	284	(4.12)	0.01	0.7019

**Table 2:** Outcomes between the two groups. HCC—hepatocellular carcinoma.

Variable	N	Exposure		Non-Exposure		*p*-Value
		n	(%)	n	(%)	
**Total**	13,792	6896	(50.00)	6896	(50.00)	
Spontaneous bacterial peritonitis	150	98	(1.42)	52	(0.75)	0.0002
Decompensation	546	396	(5.74)	150	(2.18)	<0.0001
Hepatorenal syndrome	28	18	(0.26)	10	(0.15)	0.1302
Other sequelae of chronic liver disease	41	29	(0.42)	12	(0.17)	0.0078
Ascites	342	298	(4.32)	44	(0.64)	<0.0001
Jaundice	75	54	(0.78)	21	(0.30)	0.0001
Hepatic coma	294	191	(2.77)	103	(1.49)	<0.0001
Variceal bleeding	260	197	(2.86)	63	(0.91)	<0.0001
All-cause mortality	966	577	(8.37)	389	(5.64)	<0.0001
Liver transplantation	21	6	(0.09)	15	(0.22)	0.0494
HCC	462	232	(3.36)	230	(3.34)	0.9246

**Table 3:** Factors associated with spontaneous bacterial peritonitis. CCI—Charlson comorbidity index.

Variable	Adjusted HR	95% CI		*p*-Value
Propranolol Exposure vs. Non-Exposure	1.24	(0.88)	(1.75)	0.2111
**Sex**				
Male vs. female	1.90	(1.30)	(2.76)	0.0008
**Age Group**				
18–34				
35–49	1.15	(0.66)	(2.00)	0.6204
50–64	1.34	(0.74)	(2.40)	0.3307
65+	2.02	(1.08)	(3.75)	0.0270
**Covariate**				
**CCI**				
Congestive heart failure	1.98	(0.76)	(5.11)	0.1603
Peripheral vascular disease	2.98	(0.41)	(21.83)	0.2819
Cerebral vascular accident	0.53	(0.18)	(1.61)	0.2657
Pulmonary disease	0.92	(0.52)	(1.63)	0.7716
Peptic ulcer	1.27	(0.86)	(1.88)	0.2279
Liver cirrhosis	1.02	(0.69)	(1.49)	0.9260
Diabetes	1.17	(0.61)	(2.24)	0.6318
Diabetes complications	0.57	(0.17)	(1.90)	0.3612
Paraplegia	4.72	(0.55)	(40.53)	0.1570
Renal disease	1.63	(0.70)	(3.80)	0.2605
Hypertension	0.71	(0.43)	(1.18)	0.1845
**Baseline Medications**				
Metformin	1.41	(0.67)	(2.95)	0.3676
Aspirin	0.45	(0.17)	(1.15)	0.0949
Angiotensin converting-enzyme inhibitor	0.82	(0.34)	(2.01)	0.6670
Lipid lowering drugs	1.10	(0.35)	(3.52)	0.8688
Diuretics	1.83	(0.96)	(3.47)	0.0652
Selective beta blockers	0.83	(0.34)	(2.04)	0.6904
**Concomitant Medications**				
Selective beta blockers	0.35	(0.19)	(0.67)	0.0014
Diuretics	8.56	(5.93)	(12.37)	<0.0001

**Table 4:** Factors associated with all-cause mortality.

Variable	Adjusted HR	95% CI		*p*-Value
**Propranolol Exposure**				
**1~28 Days vs. non-exposure**	1.33	(1.15)	(1.53)	0.0001
**29~90 Days vs. non-exposure**	1.12	(0.91)	(1.40)	0.2888
**>90 Days vs. non-exposure**	0.79	(0.64)	(0.98)	0.0340
**Sex**				
Male vs. female	1.59	(1.38)	(1.83)	<0.0001
**Age Group**				
18–34				
35–49	1.73	(1.34)	(2.21)	<0.0001
50–64	2.63	(2.04)	(3.40)	<0.0001
65+	5.07	(3.89)	(6.61)	<0.0001
**Covariate**				
**CCI**				
Acute myocardial infarction	2.27	(0.89)	(5.82)	0.0870
Congestive heart failure	1.37	(0.95)	(1.97)	0.0914
Peripheral vascular disease	3.15	(1.67)	(5.93)	0.0004
Cerebral vascular accident	1.56	(1.20)	(2.01)	0.0008
Dementia	1.86	(1.04)	(3.32)	0.0350
Pulmonary disease	1.25	(1.03)	(1.52)	0.0218
Connective tissue disorder	0.49	(0.16)	(1.51)	0.2133
Peptic ulcer	1.29	(1.11)	(1.50)	0.0009
Liver cirrhosis	0.78	(0.67)	(0.92)	0.0025
Diabetes	1.17	(0.92)	(1.50)	0.2098
Diabetes complications	1.21	(0.85)	(1.72)	0.2872
Paraplegia	0.75	(0.31)	(1.79)	0.5116
Renal disease	1.87	(1.42)	(2.48)	<0.0001
Severe liver disease	1.61	(0.22)	(11.63)	0.6392
Hypertension	1.06	(0.89)	(1.27)	0.5056
**Baseline Medications**				
Metformin	1.16	(0.87)	(1.55)	0.3157
Aspirin	0.84	(0.65)	(1.09)	0.1964
Angiotensin converting-enzyme inhibitor	0.71	(0.52)	(0.97)	0.0307
Lipid lowering drugs	0.80	(0.48)	(1.32)	0.3820
Statins	0.91	(0.40)	(2.04)	0.8147
Diuretics	1.88	(1.45)	(2.44)	<0.0001
Selective beta blockers	0.94	(0.70)	(1.26)	0.6631
**Concomitant Medications**				
Selective beta blockers	0.44	(0.35)	(0.56)	<0.0001
Diuretics	2.69	(2.33)	(3.11)	<0.0001

**Table 5:** Factors associated with hepatocellular carcinoma.

Variable		Adjusted HR	95% CI		*p*-Value
**Propranolol Exposure**					
**1~28 Days vs. non-exposure**		0.81	(0.65)	(1.01)	0.0580
**29~90 Days vs. non-exposure**		0.80	(0.58)	(1.09)	0.1588
**>90 Days vs. non-exposure**		0.49	(0.36)	(0.67)	<0.0001
**Sex**					
Male vs. female		1.47	(1.20)	(1.79)	0.0002
**Age Group**					
18–34					
35–49		3.33	(2.02)	(5.49)	<0.0001
50–64		7.67	(4.68)	(12.55)	<0.0001
65+		10.72	(6.43)	(17.88)	<0.0001
**Covariate**					
**CCI**					
1	Acute myocardial infarction	1.82	(0.25)	(13.18)	0.5552
2	Congestive heart failure	0.98	(0.45)	(2.10)	0.9489
3	Peripheral vascular disease	0.74	(0.10)	(5.26)	0.7597
4	Cerebral vascular accident	0.78	(0.46)	(1.33)	0.3695
6	Pulmonary disease	0.79	(0.57)	(1.10)	0.1584
7	Connective tissue disorder	0.64	(0.16)	(2.58)	0.5294
8	Peptic ulcer	1.03	(0.82)	(1.30)	0.7978
9	Liver cirrhosis	1.46	(1.19)	(1.78)	0.0002
10	Diabetes	0.75	(0.51)	(1.10)	0.1362
11	Diabetes complications	1.10	(0.62)	(1.96)	0.7375
12	Renal disease	0.90	(0.47)	(1.71)	0.7458
13	Hypertension	0.49	(0.37)	(0.66)	<0.0001
**Baseline Medications**					
Metformin		2.08	(1.40)	(3.08)	0.0003
Aspirin		0.71	(0.46)	(1.11)	0.1355
Angiotensin converting-enzyme inhibitor		1.23	(0.80)	(1.90)	0.3513
Lipid lowering drugs		0.50	(0.20)	(1.21)	0.1248
Statins		1.30	(0.41)	(4.09)	0.6527
Diuretics		0.74	(0.44)	(1.24)	0.2547
Selective beta blockers		1.21	(0.78)	(1.87)	0.3858
**Concomitant Medications**					
Selective beta blockers		0.54	(0.39)	(0.74)	0.0001
Diuretics		5.65	(4.61)	(6.92)	<0.0001

## Supplementary Materials

The following are available online at https://www.mdpi.com/1010-660X/56/1/14/s1. Table S1: Patient’s characteristics before propensity score matching. Table S2: Factors associated with decompensation. Figure S1: Cumulative incidence of decompensation between groups.
