# Supplementary material for: The Role of Non-Selective β-Blockers in Compensated Cirrhotic Patients without Major Complications
Source: Medicina (Kaunas). 2019 Dec 30;56(1):14. doi: 10.3390/medicina56010014 (PMC7022668; doi:10.3390/medicina56010014)
Supplement: Supplementary file 1 [file medicina-56-00014-s001.pdf]

Table S1. Patient's characteristics before propensity score matching.

		Before Matching			
		**Exposure**	**Non-exposure**		
**Variable**	**N**	**n (%)**	**n (%)**	**SMD**	***p*-value**
**Total**	37,246	7002 (18.80)	30,244 (81.20)		
**Sex**					<0.0001
Female	11849	2696 (38.50)	9153 (30.26)	0.17	
Male	25397	4306 (61.50)	21,091 (69.74)	0.17	
**Age group**				0.01	<0.0001
18–34	9326	1616 (23.08)	7710 (25.49)		
35–49	13,938	2787 (39.80)	11,151 (36.87)		
50–64	9186	1778 (25.39)	7408 (24.49)		
65+	4796	821 (11.73)	3975 (13.14)		
**Covariate**					
(1) Acute myocardial infarction	85	9 (0.13)	76 (0.25)	0.03	0.0524
(2) Congestive heart failure	488	68 (0.97)	420 (1.39)	0.04	0.0056
(3) Peripheral vascular disease	155	18 (0.26)	137 (0.45)	0.03	0.0217
(4) Cerebral vascular accident	1321	185 (2.64)	1136 (3.76)	0.06	<0.0001
(5) Dementia	188	20 (0.29)	168 (0.56)	0.04	0.0041
(6) Pulmonary disease	2437	457 (6.53)	1980 (6.55)	0.00	0.9513
(7) Connective tissue disorder	261	55 (0.79)	206 (0.68)	0.01	0.3455
(8) Peptic ulcer	4979	1143 (16.32)	3836 (12.68)	0.10	<0.0001
(9) Liver disease	7717	1588 (22.68)	6129 (20.27)	0.06	<0.0001
(10) Diabetes	3294	488 (6.97)	2806 (9.28)	0.08	<0.0001
(11) Diabetes complications	665	117 (1.67)	548 (1.81)	0.11	0.4221
(12) Paraplegia	145	18 (0.26)	127 (0.42)	0.28	0.0486
(13) Renal disease	860	142 (2.03)	718 (2.37)	0.24	0.0823
(15) Severe liver disease	9	4 (0.06)	5 (0.02)	0.21	0.0489
HTN	5706	1049 (14.98)	4657 (15.40)	0.01	0.3831
**Prior medication**					
**Interferon-based therapy**					
Interferon	161	23 (0.33)	138 (0.46)	0.02	0.1418
**Metformin**	2022	332 (4.74)	1690 (5.59)	0.04	0.0049
**Aspirin**	1532	291 (4.16)	1241 (4.10)	0.00	0.8415
**Angiotensin converting-enzyme inhibitor**	952	211 (3.01)	741 (2.45)	0.35	0.0071

Captopril	295	77 (1.10)	218 (0.72)		0.0013
Lisinopril	207	36 (0.51)	171 (0.57)		0.6031
Perindopril	146	26 (0.37)	120 (0.40)		0.7588
Ramipril	118	23 (0.33)	95 (0.31)		0.8472
Quinapril	54	13 (0.19)	41 (0.14)		0.3208
Benazepril	5	3 (0.04)	2 (0.01)		0.0184
Cilazapril	34	9 (0.13)	25 (0.08)		0.2520
Fosinopril	157	37 (0.53)	120 (0.40)		0.1255
**Lipid lowering drugs**	732	131 (1.87)	601 (1.99)	0.01	0.5276
Clofibrate	1	0 (0.00)	1 (0.00)		0.6304
Bezafibrate	67	13 (0.19)	54 (0.18)		0.8993
Gemfibrozil	344	79 (1.13)	265 (0.88)		0.0470
Fenofibrate	354	45 (0.64)	309 (1.02)		0.0032
Nicotinic acid	8	2 (0.03)	6 (0.02)		0.6535
Acipimox	16	6 (0.09)	10 (0.03)		0.0555
**Statins**	350	48 (0.69)	302 (1.00)	0.03	0.0144
Atorvastatin	14	0 (0.00)	14 (0.05)		0.0718
Fluvastatin	116	26 (0.37)	90 (0.30)		0.3183
Pitavastatin	3	0 (0.00)	3 (0.01)		0.4046
Rosuvastatin	193	19 (0.27)	174 (0.58)		0.0014
Simvastatin	42	5 (0.07)	37 (0.12)		0.2525
**Diuretics**	877	145 (2.07)	732 (2.42)	0.02	0.0822
Furosemide	759	125 (1.79)	634 (2.10)		0.0969
Spironolactone	267	36 (0.51)	231 (0.76)		0.0257
**Selective beta blockers**	1608	306 (4.37)	1302 (4.30)	0.00	0.8089

Table S2. Factors associated with decompensation. CI—confidence interval;

**Variable**	Adjusted HR	95% CI		*p*-value
**Propranolol exposure**				
**1~28 days vs. non-exposure**	2.34	(1.90	2.88)	<0.0001
**29~90 days vs. non-exposure**	2.16	(1.64	2.85)	<0.0001
**>90 days vs. non-exposure**	1.43	(1.09	1.87)	0.0099
**Sex**				
Male vs. Female	1.62	(1.34	1.96)	<0.0001
**Age group**				
18–34				
35–49	1.54	(1.14	2.09)	0.0045
50–64	2.14	(1.56	2.92)	<0.0001
65+	2.81	(2.00	3.96)	<0.0001
**Covariate**				
**CCI**				
Acute myocardial infarction	0.69	(0.09	5.32)	0.7203
Congestive heart failure	0.79	(0.41	1.53)	0.4809
Peripheral vascular disease	0.69	(0.10	4.98)	0.7173
Cerebral vascular accident	1.09	(0.70	1.70)	0.7090
Dementia	2.12	(0.78	5.75)	0.1386
Pulmonary disease	0.77	(0.56	1.06)	0.1058
Connective tissue disorder	0.28	(0.04	2.01)	0.2058
Peptic ulcer	1.57	(1.29	1.91)	<0.0001
Liver disease	0.94	(0.77	1.15)	0.5597
Diabetes	0.75	(0.52	1.08)	0.1214
Diabetes complications	1.09	(0.65	1.82)	0.7574
Paraplegia	1.51	(0.34	6.69)	0.5889
Renal disease	0.99	(0.59	1.67)	0.9798
Hypertension	0.67	(0.51	0.88)	0.0041
**Baseline medications**				
**Metformin**	1.91	(1.29	2.83)	0.0011
**Aspirin**	0.65	(0.42	1.00)	0.0491
**Angiotensin converting-enzyme inhibitor**	0.68	(0.42	1.10)	0.1165
**Lipid lowering drugs**	1.19	(0.66	2.13)	0.5675
**Diuretics**	2.67	(1.91	3.73)	0.0000
**Selective beta blockers**	1.46	(0.96	2.22)	0.0784
**Concomitant medications**				

**Selective beta blockers**	0.28	(0.20 0.41)	<0.0001
**Diuretics**	5.09	(4.21 6.16)	<0.0001

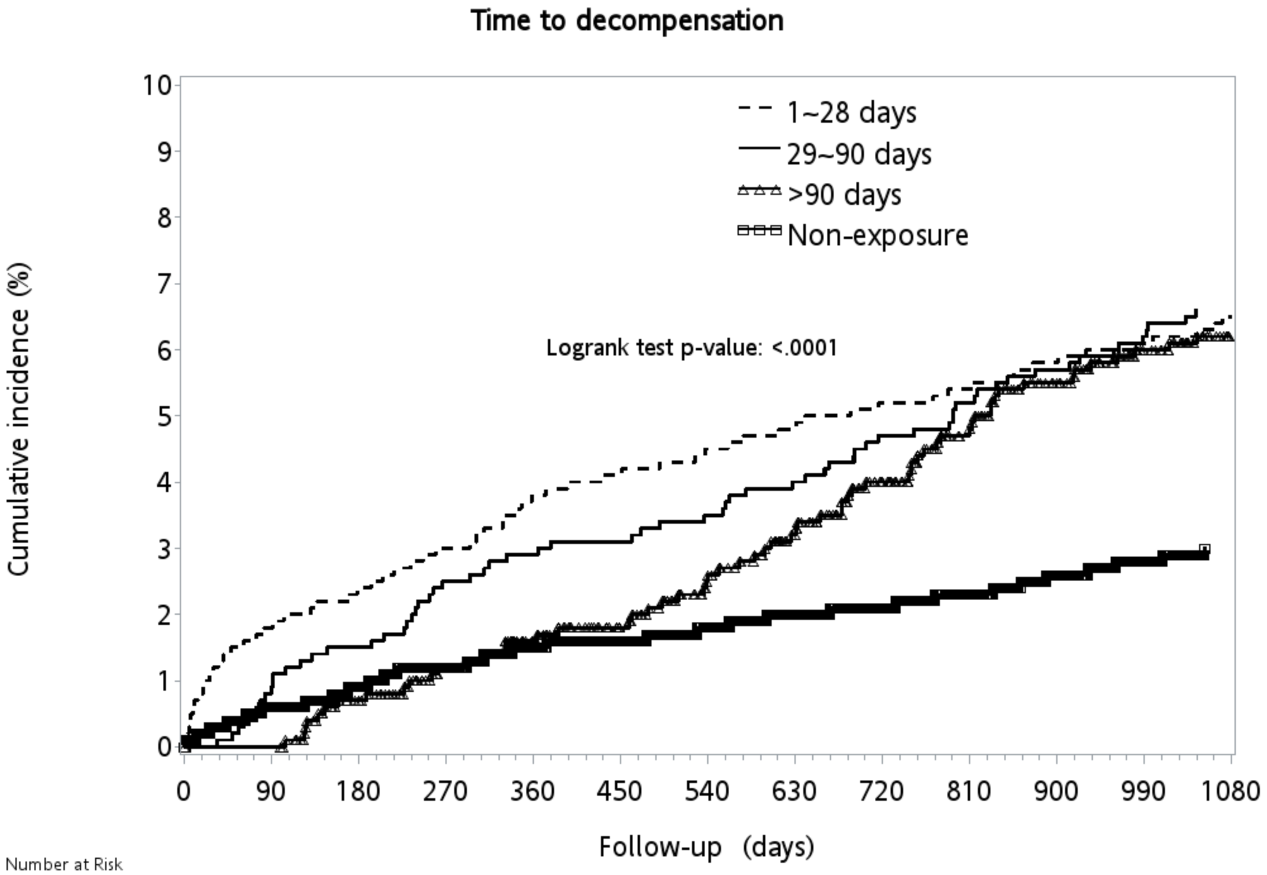

Number at Risk													
Non-exposure	6896	6331	5852	5458	5094	4799	4495	4220	3948	3712	3482	3285	3101
1~28 days	4028	3789	3664	3551	3441	3328	3234	3131	3021	2908	2807	2719	2612
29~90 days	1358	1309	1262	1222	1190	1155	1117	1087	1049	1013	983	953	918
>90 days	1510	1510	1481	1446	1407	1372	1339	1309	1262	1218	1183	1140	1105

Figure S1. Cumulative incidence of decompensation between groups.
